# Effect of Different Light Qualities and Intensities on the Yield and Quality of Facility-Grown *Pleurotus eryngii*

**DOI:** 10.3390/jof8121244

**Published:** 2022-11-24

**Authors:** Zonghan Yue, Wei Zhang, Wenjun Liu, Jia Xu, Wen Liu, Xinyu Zhang

**Affiliations:** 1 Xiong’an Institute of Innovation, Baoding 071700, China; 2 Department of Optics and Optical Engineering, University of Science and Technology of China, Hefei 230026, China

**Keywords:** *Pleurotus eryngii*, light, cultivation, nutrition quality, yield

## Abstract

Proper light is essential for the formation and development of macrofungi fruiting bodies. Currently, there are unclear treatment conditions, such as light quality and light intensity, in the production of *Pleurotus eryngii* in intensive cultivation facilities, which is not helpful to the formation and implementation of standardized production programs. The research discussed in this paper investigated the effects of different light quality and intensity conditions on the yield and quality of *P. eryngii*. The results showed that the yield and nutritional quality of the red light treatment samples were higher than those of the white light control, the commercial properties were good, and the energy consumption of the red LED light source was the lowest under the same light intensity. The results of this experiment further provide a reference for the energy-saving and high-quality cultivation of *P. eryngii*.

## 1. Introduction

Light, as an important environmental factor, plays an important regulatory role in the growth, development and physiological metabolism of organisms. Light can provide a source of energy for plant photosynthesis [1], and it also plays an important role in regulating the growth and development of fungi and the metabolism of secondary substances as an external environmental signal [2,3].

Over the years, many scholars have studied the regulatory effects of light on fungal vegetative growth, sexual development and anabolism of natural products. Many studies have shown that light treatment affects the asexual growth of macrofungal mycelium, and further studies have found that different light quality treatments affect the growth of mycelium differently. Previous studies showed that treatment with higher intensity fluorescent light sources strongly inhibited the growth of mono- and binucleate mycelium of *Pleurotus ostreatus* [4]. Further tests showed that blue light significantly inhibited the ability of the apical mycelia of *Tuber borchii* to branch, as its colonies were thinner and less apically branched compared to those in the dark [5]. The effect of different light qualities on the mycelial growth of *Cordyceps militaris* was different; the growth rate of mycelia under white light treatment was faster than under dark treatment, while blue light significantly inhibited its colony growth. However, the aerial mycelia density of all strains treated under different light qualities was significantly lower than the aerial mycelial density under dark conditions [6].

For most macrofungi, light is one of the environmental conditions necessary to induce primordia differentiation and to regulate the normal growth of fruiting bodies.

Light treatments can make *P. ostreatus* [7] and *Coprinus* sp. [8,9] and other basidiomycetes primordia to differentiate normally into well-developed fruiting bodies. On the other hand, the primordia of samples under dark treatment were unable to develop normally such that the basidium could not undergo normal nucleation and therefore the samples could not complete their sexual life history. Light is also essential for the normal differentiation of ascomycetes stromata such as *C. militaris* [10].

Light is one of the essential environmental conditions for the differentiation and further development of *Pleurotus sajor-caju* and *Volvariella volvacea* fruiting bodies [11]. However, different light source irradiation treatments had differential effects on the differentiation of macrofungal fruiting bodies. Yellow light of a certain intensity had a strong inductive effect on the formation of primordia of *Sparassis Crispa* [12]. Blue light had an important role in promoting the formation of *Schizophyllum commune* primordia and the development of fruiting bodies [13]. Blue light was also important for the maturation of the primordia of *Lentinula edodes*, which are darker in color [14,15]. The effects of different light treatments on *Hypsizygus marmoreus* primordia formation were significantly different, with slower cap differentiation in red light and dark treatments and a faster cap differentiation in green light [16].

Light treatment accelerated the growth of *Alnicola lactariolens* and *Hebeloma vinosophyllum* fruiting bodies. While the stipe length became shorter and larger in diameter as the light intensity gradually increased, the cap diameter of both fungi differed with light intensity, with the cap diameter increasing and the stipe length decreasing as the light duration increased [17].

Light treatment can regulate the content of macrofungal metabolites, and light is essential for carotenoid synthesis in *C. militaris*. The carotenoid content was significantly higher under blue light than under natural light [18,19]. White light favored the accumulation of cordycepin in the stromata of *C. militaris*, but the effect of different light conditions on the adenosine content was not significant [20]. Light also regulated the production of *Isaria farinose* pigments [21], with a red light treatment showing the greatest activity of *L. edodes* mycelium extracellular enzymes. Green and blue light treatments had a positive effect on the accumulation of polysaccharides, soluble proteins and polyphenols [22]. Light treatment promoted the production of L-ascorbic acid-like substances in the mycelia of *P. ostreatus*, which also induced the formation of primordia to some extent [7].

*Pleurotus eryngii* var. eryngii is a well-known edible fungus that is widely distributed in the subtropical Mediterranean region as well as in central Europe and Asia [23]. With its soft texture and unique almond-like flavor, it is favored by edible mushroom lovers and gourmets around the world and has a promising market. It is rich in protein, polysaccharides, fiber and other nutrients [24,25].

Studies have shown that *P. eryngii* can treat hyperlipidemia [26], have antiviral [27] and anticancer [28] properties and enhance immune function [29], making it highly valuable for medicinal purposes.

In recent years, as the factory cultivation model of *P. eryngii* continues to spread, there has been an increasingly urgent demand for research on standardized cultivation techniques. At present, artificial light sources, mainly fluorescent lamps, are widely used for mushroom induction in factory mushroom production, but there is a general lack of clarity in light quality and light intensity in the management process. This is not helpful for forming and implementing standardized production schemes. Compared to fluorescent lamps, light emitting diodes (LEDs) are more energy efficient and consume less energy, have a longer service life, have a wider wavelength range and can be adjusted in light intensity. At the same time, LEDs generate less heat than commonly used artificial lighting sources, which facilitates the precise regulation and maintenance of the biological culture environment. The researchers also estimated the power conversion efficiency of LED systems for plant light supplementation, showing that LED systems can be twice as efficient as ordinary fluorescent lighting systems [30]. The results also demonstrate the potential of LED light sources for high-quality, energy-efficient production of mushrooms.

At present, the factory production of *P. eryngii* still relies heavily on the previous management experience of cultivation technicians for light management, and there is little research on the selection of light quality and the optimum light intensity for the management of *P. eryngii* during the mushroom emergence period.

In this research, LED light sources were selected to supplement the light in the production of *P. eryngii*. By investigating the effects of different light qualities and light intensities on the yield, appearance quality and nutritional quality of *P. eryngii*, the aim was to identify the most suitable light quality and optimal light intensity for the management of the mushroom production period in the factory. Furthermore, the goal was to provide a reference basis and technical support for the energy-saving and high-quality cultivation of *P. eryngii* in the factory.

## 2. Materials and Methods

The *P. eryngii* test strain “Fengyuan No.2” was provided by Anhui Fengyuan Food Co., Bengbu, China, The cultivation substrate consisted of 35% corncob, 23% poplar sawdust, 10% maize meal, 10% soybean meal, 20% wheat bran, 1% lime and 1% gypsum, with a water content of 65%. The substrate was mixed thoroughly using a stirring device, and then the prewetted substrate was packed into polypropylene cultivation bags weighing 1450 g each and sterilized at 0.125 KPa and 121 °C for 2 h. After cooling at room temperature in an aseptic environment, the cultures were inoculated with the *P. eryngii* strain. After inoculation, the bags were placed in a dark culture room at a constant temperature of 25 °C until the medium was completely covered with mycelia, and cultivation bags were transferred to a culture room at 15 °C for 7 d, after which they were cultivated under different treatments of light conditions. All the cultivation bags were placed in a mushroom culture room with a carbon dioxide detection system and automatic humidification and ventilation system to maintain suitable environmental conditions for the growth of *P. eryngii*. The culture room was equipped with some cultivation racks to facilitate the arrangement of cultivation bags. We always maintained 85% air humidity in the growth management. To better promote the primordia formation, the CO_2_ concentration in the culture room was kept at 3000 ppm at the beginning of the primordia formation. Thereafter, the CO_2_ concentration was adjusted to about 1200 ppm after 2–3 days, according to the actual situation observed, until they had matured. The ventilation system was automatically turned on by the CO_2_ concentration monitoring equipment in the culture room. These environmental conditions are widely used in the cultivation of *P. eryngii*.

**Figure 1:**
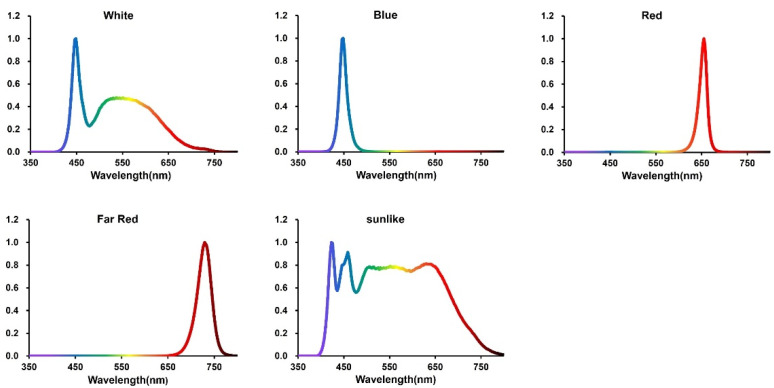
Spectral maps of the five light treatments of the experiment.

During the previous research experiments, we found that the strains of *P. eryngii* could not differentiate into normal primordia under the dark treatment [31], so five light qualities (CK, B, R, FR, sunlike) were used in this experiment, and the specific information on the spectral characteristics of the different light treatments is shown in Table 1 and Figure 1.

Cultivation bags treated with different light conditions were placed horizontally on the cultivation racks in different groups, so that the growing side of the fruiting bodies faced the light source. Twenty-five LED light sources (LED-08-28400, DEVOTION, Fuzhou, China) were fixed on the opposite side of the cultivation racks so that the light emitted from them was perpendicular to the growing side of the cultivation bags. A light sensor logger (LI-1500, LI-COR, Lincoln, NV, USA) was used to measure the light quantum flux density on the surface of the growing side of the bags in real time, while adjusting the light intensity of the light source to meet the conditions required for the experiment. Five different light qualities and five different intensities were used, and a total of 25 experimental treatment areas were applied separated from each other by opaque shade cloth so that each treatment area was not disturbed by other conditions. The light treatment was carried out until the end of harvest, with 12 h of light treatment per day. With reference to the actual conditions of light distribution and light quantum flux in the light treatment in the cultivation of *P. eryngii*, five different light intensity gradients were set, 1–2 μmol.m^−2^.s^−1^, 2–3 μmol.m^−2^.s^−1^, 3–4 μmol.m^−2^.s^−1^, 4–5 μmol.m^−2^.s^−1,^ and 5–10 μmol.m^−2^.s^−1^. This experiment was conducted in the same culture room with a total of 25 experimental treatment areas, and 10 cultivation bags were placed in each treatment area.

In the process of cultivation, and according to the cultivation practices of primordia formation, only 3 strong primordia were chosen to continue cultivation. Mature fruiting bodies were harvested according to the cultivation practices, and their weight was measured. The ratio between the diameter of the cap and stipe was calculated, the growth form of fruiting bodies was evaluated, and the yield of fruiting bodies from each bag was counted. 

To study the effects of different light quality treatments on the nutritional quality of the fruiting bodies of *P. eryngii,* we collected 25 samples of them under different light conditions treatments. For each treatment, five mature fruiting bodies with the same light quality and different light intensities were selected, and the fresh mushrooms were sliced using a sterilized blade, then dried, pulverized and mixed well, screened with a 40-mesh size, and weighed precisely to 100 g subjected to nutrient content determination. The total protein content of the powdered fruiting bodies was determined using the Kjeldahl method with the nitrogen/protein conversion factor of 4.38 [32]; the amino acid content was determined by the acid hydrolysis of amino acids; the water-soluble polysaccharides were separated by hot-water extraction [33]. Finally, the content was measured by the phenol sulfate method [34]. In each of the above experiments, three biological replicates were performed for each treatment. Parameters such as the ratio of essential amino acids to total amino acids were calculated from the relevant data; amino acid scores and essential amino acid indices were calculated using the relevant formulas [35].

Data were analyzed using Excel 2010 and SPSS statistical software, and figures were produced using GraphPad Prism.

## 3. Results

### 3.1. Analysis of the Interaction Effects of Different Light Qualities and Light Intensities on the Yield of P. eryngii

A univariate multifactor ANOVA was used to analyze whether there was an interaction between different light qualities and different light intensities on the yield of a single bag of *P. eryngii*, the results of which are shown in Table 2.

By comparing the F value and sig. value of light quality, light intensity and light quality * light intensity, the F value of light quality was the largest and the sig. value was the smallest, with sig. < 0.05, while the sig. values of light intensity and light quality * light intensity were less than 0.05; the main effects of light intensity and light quality reached significance, and the interaction of light quality * light intensity also reached significance. Therefore, there was an interaction between different light qualities and different light intensities on the yield of *P. eryngii*. A simple effect evaluation was needed to further analyze the effect of different light quality treatments on the yield of *P. eryngii*. It had to be done in the same light intensity range and the effect of different light intensity treatments on the average yield each bag had to be explored under the same light quality conditions.

### 3.2. Effect of Different Light Quality Treatments on the Yield of P. eryngii in the Same Light Intensity Range

**Figure 2:**
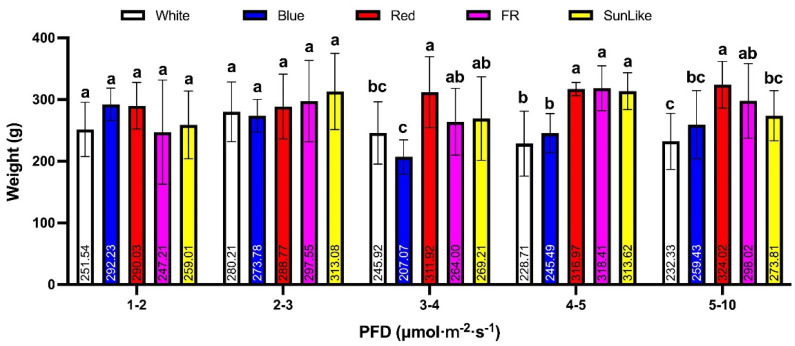
Effect of different light quality treatments on the yield of the fruiting bodies of *P. eryngii* in the same light intensity range. Different lowercase letters marked in the table indicate significant differences between treatments (*p* < 0.05). The same below.

Based on the results of the analysis of different light quality treatments in the same light intensity range shown in Figure 2, there was no significant difference in yield between different light quality treatments and the white light control in the light intensity range of 1–2 and 2–3 μmol.m^−2^.s^−1^, However the yield was significantly higher under the red light treatment compared to the white light control in the light intensity range of 3–4, 4–5 and 5–10 μmol.m^−2^.s^−1^, and the yield was significantly higher under the red light treatment than the white light control in the light intensity range of 4–5 μmol.m^−2^.s^−1^. In the range of 4–5 μmol.m^−2^.s^−1^, yields were significantly higher under the sunlike and far-red light treatments than under the white light control; the blue light treatment did not show any significant difference in the yield of the *P. eryngii* fruiting bodies under the five different light gradients compared to the white light control.

The average yield of *P. eryngii* under red light irradiation at 5–10 μmol.m^−2^.s^−1^ was the highest of all samples at 324.02 g.

**Figure 3:**
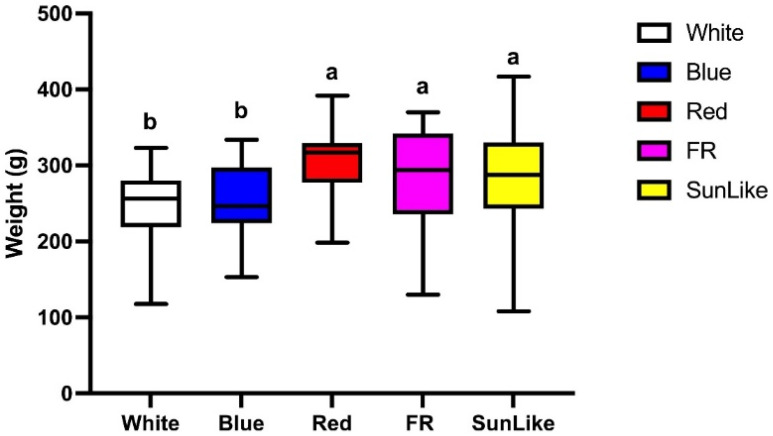
Effect of different light quality light treatments in the range of 1–10 μmol.m^−2^.s^−1^ on the yield of fruiting bodies of *P. eryngii*.

In order to simulate the actual situation in production, considering the problem of uneven light in the actual production of *P. eryngii*, a statistical analysis was conducted on the yield of *P. eryngii* fruiting bodies with different light quality treatments in the range of 1–10 μmol.m^−2^.s^−1^. The effect of different light quality treatments on the *P. eryngii* fruiting bodies was more obvious, and the *P. eryngii* fruiting bodies under red light, sunlike and far-red light treatments were significantly higher than those under white light treatment, based on the results shown in Figure 3.

### 3.3. Effect of Different Light Intensity Treatments with the Same Light Quality on the Yield of P. eryngii

**Figure 4:**
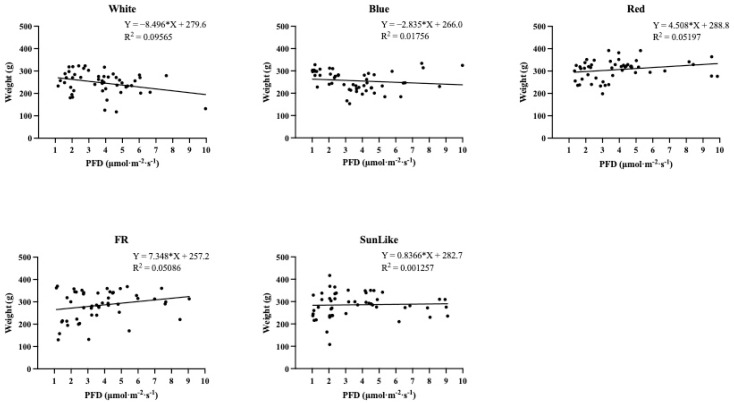
Effect of different light intensity treatments with the same light quality on the yield of the fruiting bodies of *P. eryngii*.

According to Figure 4’s analysis of the impact of different light intensities on the average yield of *P. eryngii* fruiting bodies per bag for the same light quality, higher light intensities were not beneficial for increasing the overall yield per bag under the white and blue light treatments. A higher light intensity, however, had a positive effect on the increase of total yield per bag of *P. eryngii* fruiting bodies under sunlike, red, and far-red light treatments. 

### 3.4. Effect of Different Light Qualities and Light Intensities on the Diameter Ratio of the Cap and Stipe of P. eryngii

A univariate multivariate ANOVA was used to analyze whether there was an interaction between different light qualities and different light intensities on the cap and stipe diameter ratio of the *P. eryngii* fruiting bodies. The results are shown in Table 3.

By comparing the F values and sig. values for light quality, light intensity and light quality * light intensity, it is clear that the sig. for light quality was less than 0.05, while the sig. values for light intensity and light quality * light intensity were both greater than 0.05.

In summary, the main effect of light quality reached significance, while the light intensity and light quality * light intensity interactions did not reach significance. Therefore, there was no significant interaction between light quality and light intensity on the cap and stipe diameter ratio of *P. eryngii* fruiting bodies. The effect of light intensity on the cap and stipe diameter ratio of the *P. eryngii* fruiting bodies was not significant. Therefore, a further analysis of the effect of different light quality treatments on the cap and stipe diameter ratio of *P. eryngii* fruiting bodies is needed.

From Table 4, it can be seen that the largest cap and stipe diameter ratio was found in the blue light treatment with an average of 1.28, which was significantly larger than the white light treatment; the smallest ratio was found in the far-red light treatment with an average of 1.16. This was significantly smaller than that of the white light treatment, and there was no significant difference between the ratio in the red light and sunlike light treatments and the white light control.

### 3.5. Effect of Different Light Quality Treatments on the Nutritional Quality of P. eryngii Fruiting Bodies

From Table 5, among the five light treatments, the water-soluble polysaccharides content of the samples under the white light treatment was the lowest at 5.35 g/100 g. This indicated that all four light treatments except white light were beneficial to the increase in water-soluble polysaccharides content in the P. eryngii fruiting bodies, with the water-soluble polysaccharides content of the samples under the red light treatment the highest at 6.53 g/100 g. The protein content of the samples under white light treatment was the highest at 15.96 g/100 g, indicating that all four light treatments except white light were not helpful for the accumulation of protein in the P. eryngii fruiting bodies. The protein content of the samples under red light treatment was the lowest at 10.53 g/100 g.

The total content of the 16 amino acids was also affected by the different light treatments. The highest content was found in the samples under sunlike light treatment with 13.87 g/100 g, followed by white light treatment, and the lowest content was found in the samples under far-red light treatment with 10.12 g/100 g. The total amount of the 16 amino acids was only one of the factors used to measure the nutritional quality of *P. eryngii* fruiting bodies. Therefore, we further analyzed the amino acid composition of the proteins in the tested samples as a basis for assessing the nutritional value of the proteins in the different samples.

The results of the analysis of the amino acid composition and content of the proteins of the *P. eryngii* fruiting bodies under different light quality treatments are shown in Table 6.

The ratio of various amino acid compositions in the protein of *P. eryngii* fruiting bodies was basically similar. The most abundant amino acid was glutamic acid, followed by aspartic acid, both of which are umami taste amino acids. In addition to these two amino acids, glycine and alanine are also umami taste amino acids, and the content of these amino acids mainly determines the degree of umami taste of the samples. We compared the umami taste amino acid content in the samples under different light treatments, and the results showed that the UAA/TAA of the samples under red light treatment was the lowest, at 38.18%. The UAA/TAA of the treatment samples were all lower than that of the white light control.

Essential amino acids are amino acids that are entirely dependent on external intake and cannot be synthesized by the human body, and their content determines the nutritional value of the protein. To measure the nutritional value of the samples under different light treatments, we compared the EAA/TAA of the samples. We showed that it ranged from 39.68% to 41.88% and the EAA/NEAA ranged from 65.78% to 72.06% for all five light treatments. Both ratios were higher than the FAO/WHO model reference values for all five treatments. The two ratios showed the same trend under different light quality treatments, with both ratios being highest in the red light treatment and lowest in the sunlike light treatment. Both ratios were higher in the white light treatment than in the blue light and sunlike light treatments but lower than in the red light and far-red light treatments.

Amino acid scores and essential amino acid indices were calculated based on the essential amino acid content of the proteins of *P. eryngii* fruiting bodies, and the results are shown in Table 7.

The amino acid score was used as a criterion to evaluate the samples under five different light quality treatments. The first limiting amino acid was leucine and the second limiting amino acid was isoleucine. The EAAI of the samples under the five light treatments ranged from 80.16% to 96.43%; according to the evaluation criteria, the protein quality was categorized as “high” (EAAI > 0.95), “good” (0.86 < EAAI ≤ 0.95), “useful” (0.75 < EAAI ≤ 0.86) and “inadequate” (EAAI ≤ 0.75). Based on the above, the *P. eryngii* fruiting bodies samples under the red light treatment were high protein sources, the samples under the blue and far-red light treatment were good protein sources and the remaining two samples were all useful protein sources.

### 3.6. Calculation of Energy Consumption of LED Light Sources with Different Light Qualities

The power of LED lamps of different light qualities at a light intensity of 10 μmol.m^−^^2^. s^−^^1^ was tested using a power detector, and the results are shown in Table 8.

A comparison of the measured power of different light quality LED sources at an illumination intensity of 10 μmol.m^−^^2^. s^−^^1^ showed that red LEDs consumed the least energy at the same light intensity, while blue LEDs consumed slightly less power compared to the white control, sunlike LEDs consumed the same power as the white control; and far-red LEDs consumed the most energy.

## 4. Discussion

The results of this study showed that there was an interaction between different light qualities and different light intensities on the yield of *P. eryngii* fruiting bodies, and both environmental conditions were closely related to the yield.

This experiment investigated the effect of different light intensities on the average yield each bag of *P. eryngii* under the same light quality irradiation conditions. The lighting was often uneven. In reference to the findings of previous studies, we found that in a large number of trials, researchers tended to use lux as the unit of light intensity to describe light intensity [7,16]. However, the wavelengths and energies of the components of different light sources often vary greatly, thus using lux as the unit introduces descriptive errors that are not conducive to an accurate description of light intensity.

With reference to the measurement of light intensity in plant production studies, we used a photon flux meter to measure the light intensity in different areas of the cultivation frame in *P. eryngii* production rooms. We refer to this as the photon flux density (PFD) in μmol.m^−2^. s^−1^. After extensive field investigations, we obtained a maximum light flux of 4–5 μmol.m^−2^.s^−1^ on the surface of the mushroom in production. The actual measured light intensity in most areas was below 4–5 μmol.m^−2^. s^−1^. Therefore, we added a higher light intensity of 5–10 μmol.m^−2^. s ^−1^, which exceeded the usual production values, to investigate the effects of a higher light intensity on the production of the mushroom. We therefore increased the light intensity to a higher value than the usual one to investigate the effect of a higher light intensity on the indicators in the production of *P. eryngii*.

The results of this investigation showed that the yield of *P. eryngii* gradually decreased with increasing light intensity under white and blue light treatments. Under sunlike and far-red light treatments, the yield of *P. eryngii* showed an overall increasing trend with the increasing light intensity. However, the increase was not significant, while the yield of *C. militaris* showed an overall significant increasing trend with the increasing light intensity under red light treatment. Chao et al. [36]. showed that the yield of *C. militaris* gradually increased with an increasing light intensity. This is also in line with some of the findings of our present study.

The effect of different light quality light treatments on the average yield of *P. eryngii* in each bag was more obvious. The average yield of each bag under red, sunlike and far-red light treatments was significantly higher than that under the white light control treatment. Red light could significantly promote the growth of *P. eryngii* [37]; this is also consistent with the findings of the study in which *C. militaris* [20] and *Flammulina velutipes* [38] were grown and also consistent with the findings of Kim et al. [37].

Although the average yield of a single bag of *P. eryngii* under blue light treatment was slightly higher than the average yield of a single bag under white light control, the difference between them was not significant. In addition, blue light was also reported to promote the growth of *Pleurotus nebrodensis* [39] and *C. militaris* [19], but not significantly compared to the control, which is similar to the findings of this study.

**Figure 5:**
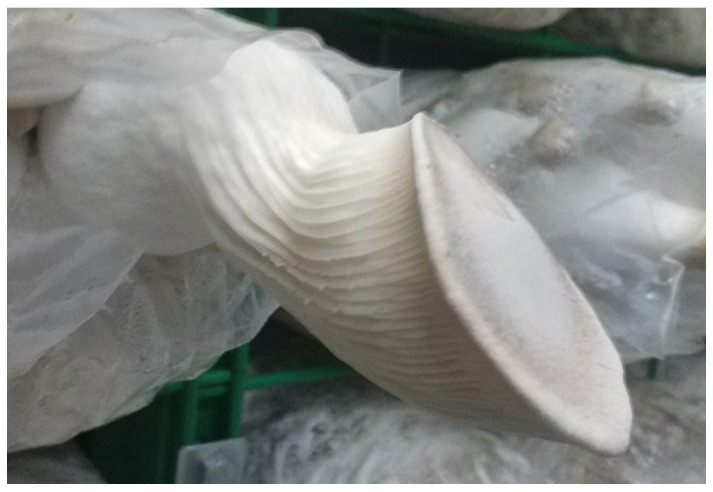
Fruiting body of *P. eryngii* under blue light irradiation treatment.

In addition to yield, different light conditions can also affect the appearance of *P. eryngii* fruiting bodies. Closer values of cap diameter to stipe diameter can indicate a more aesthetically pleasing appearance that is more desirable for the market. These experiments investigated the effect of different light qualities and light intensities on the diameter ratio of the cap and stipe of the mushroom. This study found that blue light significantly increased the diameter ratio of the cap and stipe in *P. eryngii*, as shown in Figure 5. The same conclusion was also found in the study of *Pleurotus* sp. [40,41,42].

The diameter ratio of the cap and stipe in the red light and sunlike light treatments was not significantly different from that in the control treatment, and they had a better morphological appearance and better growth consistency. The ratio in the far-red light treatment was significantly smaller than that in the control treatment, indicating that far-red light controlled the development of the cap to a certain extent. At the same time, it was also found that the different light irradiation treatments caused some changes in the length of the stipe of the mushroom. Red light caused stipes to elongate and blue light to shorten their length. This phenomenon was also observed in the studies of *P. eryngii* [31,43], *H. marmoreus* [16] and *L. edodes* [44].

In this study, we also analyzed the nutritional quality of *P. eryngii* fruiting bodies under different light treatments, and the results showed that the water-soluble polysaccharides content was the lowest, but the protein content was the highest under white light treatment. The water-soluble polysaccharides content of the sample was the highest under red light treatment, and this conclusion was consistent with that of Hu et al. [45].

However, the protein content of *P. eryngii* fruiting bodies under red light treatment was the lowest, a result contrary to that of Zhang et al. [43]. Ai performed RNA-seq analysis on the dark- and light-treated *P. eryngii* fruiting bodies. The results of a KEGG metabolic pathway analysis showed that the expression of genes related to the metabolic pathways of glycine, serine and threonine changed with different light treatments [31]. This demonstrated further that light affects protein synthesis and energy metabolism during growth and is crucial for the formation and development of *P. eryngii* fruiting bodies. The results of this study showed that the ratio of fresh amino acids to total amino acids, the ratio of essential amino acids to total amino acids and the ratio of essential amino acids to nonessential amino acids were all the highest in the red light treatment. This proved to a certain extent that different light treatments affected the synthesis and metabolism of amino acids during the developmental stage of the *P. eryngii* fruiting bodies. Beta-glucan is a fiber-type complex sugar (polysaccharide) derived from the cell wall of many mushrooms. It also appears to stimulate immune function in various ways and evidence suggests it might strengthen the immune system, which would potentially benefit a wide range of conditions. Chitin is a structural polymer which is made from smaller monomers or monosaccharides and structural polymers form strong fibers. We plan to analyze the chitin content of samples treated with different light conditions in future studies. This will also help us to obtain the more accurate nitrogen/protein conversion factors. In future studies, we plan to use a textural polyhedral analysis to determine relevant textural parameters, as well as to detect chitin and glucan contents in different treatment samples. This would allow us to investigate in more depth the potential effects of different wavelengths of light conditions on the quality traits of edible mushrooms. In addition, we also plan to measure the hardness of mushrooms under different light conditions using a hardness meter, extract the relevant pigments and perform a content determination to further investigate the effect of different light conditions treatments on some important quality attributes.

Through this research, the team was able to understand the production process and growth habits of *P. eryngii*, as well as the ideal range of optimum light quality and light intensity for it. During the growth process, the results of the different indicators showed a clear pattern of light quality on the nutritional value and growth and development of the mushroom. However, the principle of light quality on the growth and development and physiological metabolism of the mushroom was not addressed in this research. The existence of red-light-receptor-related genes in fungi has been demonstrated, and the molecular mechanisms involved have been reported in studies of filamentous fungi. However, no in-depth studies of red light receptors in large fungi, especially edible fungi, have been reported so far. In our next experiments, we plan to further investigate the molecular mechanisms by which different wavelengths of light regulate the growth, development and physiological metabolism of the mushroom through transcriptomics and metabolomics. This way we can further explain how the use of light can more precisely regulate the production of the mushroom. The study will also further investigate the molecular mechanisms by which different wavelengths of light regulate growth and physiological metabolism.

In this experiment, different light qualities and light intensities were used to screen the optimum light formula for the production of *P. eryngii*. The clear conclusion was that red light was the optimum light formula for production. However, further research on photoperiod and other factors is needed at a later stage. The next step is to study different optimum light formulations according to different reproductive stages, such as special light formulations for the primordia formation stage and optimum light formulations for the rapid development stage of fruiting bodies. We also plan to study the effect of simultaneous combination of multiple light sources (e.g., red light + blue light, etc.) on the development of *P. eryngii* mushroom, commitment to better provide theoretical support for the efficient, energy-saving and high-quality production of *P. eryngii*.

## 5. Conclusions

Combining the effects of different light qualities and different light intensities on the single bag yield, cap and stipe diameter ratio and physiological and biochemical indicators such as protein, polysaccharides and protein-hydrolyzed amino acids of the *P. eryngii* fruiting bodies, the following conclusions can be drawn.

1. The average yields of single cultivation bags of *P. eryngii* fruiting bodies under red light, sunlike and far-red light treatments were all significantly higher than those under white light treatment. The average yield of a single bag under blue light treatment was slightly higher than the average yield of a single bag under white light control, but the difference between the two was not significant.
2. We conducted a statistical analysis of the total yield each bag of *P. eryngii* fruiting bodies under different light intensity treatments of the same light quality. We found that under white and blue light treatments, a higher light intensity was not conducive to the increase of the total yield of each bag of *P. eryngii* fruiting bodies. However, under sunlike, red and far-red light treatments, a higher light intensity had a positive effect on the increase of the total yield of each bag of *P. eryngii* fruiting bodies.
3. The effect of light intensity on the diameter ratio of the cap and stipe of the *P. eryngii* fruiting bodies was not significant. The diameter ratio of the cap and stipe of the *P. eryngii* fruiting bodies was the greatest, and significantly greater than the control, in the blue light treatment. The ratio was the smallest, and significantly less than the control, in the far-red light treatment. However, the difference between this ratio and that of the control was not significant in the red and sunlike light treatments.
4. According to the results of the nutritional quality analysis, the highest water-soluble polysaccharides content of *P. eryngii* fruiting bodies was under red light treatment, the lowest content was under white light control treatment. The highest protein content under white light control treatment, although the lowest protein content was under red light treatment. The UAA/TAA, EAA/TAA and EAA/NEAA amino acid scores of *P. eryngii* samples under red light treatment were the highest. The ratios of all five light treatments were higher than the FAO/WHO model reference values; based on the essential amino acid index, it was concluded that the samples under the red light treatment were high protein sources. The samples under the blue and far-red light treatment were good protein sources, and the remaining two samples were all useful protein sources. Based on the above conclusions, it can be argued that red light can optimize the amino acid nutritional value of *P. eryngii* fruiting bodies.

Therefore, red light was determined to be the most suitable light quality for the production of *P. eryngii* fruiting bodies, and the optimum light intensity for the red light treatment was 5–10 μmol.m^−2^. s ^−1^.

## Figures and Tables

**Table 1:** Information on the five light treatments of the experiment.

Processing	Spectral Characteristics
White light (CK)	4500 K white light
Blue light	450 nm ± 15 nm
Red light	660 nm ± 15 nm
Far-red light	735 nm ± 15 nm
Sunlike	Sunlike white light

**Table 2:** Examination of the intrasubject effects of different light qualities and light intensities on the yield of *P. eryngii*.

Source	Type III Sum of Squares	df	Mean Square	F	Sig.
Corrected model	243,742.550 ^a^	24	10,155.940	4.212	0.000
Intercept	1.906 × 10^7^	1	1.906 × 10^7^	7904.383	0.000
Light quality	115,596.884	4	28,899.221	11.987	0.000
Light intensity	31,223.768	4	7805.942	3.238	0.013
Light quality * light intensity	96,921.898	16	6057.619	2.513	0.001
Error	542,459.380	225	2410.931		
Total	1.984 × 10^7^	250			
Corrected Total	786,201.930	249			

Note: Dependent variable: yield, ^a^. R-squared = 0.310 (adjusted R-squared = 0.236); df: degree of freedom; F: statistics for F-Test; Sig.: significance. The same below.

**Table 3:** Examination of the intrasubject effect of different light qualities and light intensities on the diameter ratio of the cap and stipe of *P. eryngii*.

Source	Type III Sum of Squares	df	Mean Square	F	Sig.
Corrected model	0.745 ^a^	24	0.031	3.234	0.000
Intercept	366.751	1	366.751	38,218.837	0.000
Light quality	0.421	4	0.105	10.969	0.000
Light intensity	0.066	4	0.017	1.724	0.146
Light quality * light intensity	0.258	16	0.016	1.679	0.052
Error	2.159	225	0.010		
Total	369.655	250			
Corrected Total	2.904	249			

Note: Dependent variable: Diameter ratio, ^a^. R-squared = 0.257 (adjusted R-squared = 0.177).

**Table 4:** Effect of different light quality treatments on the diameter ratio of the cap and stipe diameter ratio of the *P. eryngii* fruiting bodies.

Light Quality	Diameter Ratio of the Cap and Stipe
White light	1.22 ± 0.1 b
Far-red light	1.16 ± 0.06 c
Sunlike	1.19 ± 0.14 bc
Red light	1.21 ± 0.07 b
Blue Light	1.28 ± 0.11 a

Note: Different lowercase letters marked in the table indicate significant differences between treatments (*p* < 0.05). The same below.

**Table 5:** Comparison of nutritional quality (in dry weight/100 g) of the *P. eryngii* fruiting bodies under different light quality treatments.

Light Quality	Water-Soluble Polysaccharides/g	Protein/g	Total of 16 Amino Acids/g
White light	5.35 ± 0.10 c	15.96 ± 0.42 a	13.27 ± 0.06 b
Blue Light	5.72 ± 0.12 b	14.30 ± 0.14 c	13.17 ± 0.15 b
Red light	6.53 ± 0.12 a	10.53 ± 0.18 e	10.30 ± 0.1 c
Far-red light	6.43 ± 0.09 a	11.35 ± 0.25 d	10.12 ± 0.14 c
Sunlike	5.46 ± 0.11 c	15.42 ± 0.14 b	13.87 ± 0.31 a

**Table 6:** Comparison of amino acid composition and content in proteins of *P. eryngii* fruiting bodies under different light quality treatments.

Amino Acids/%	White Light	Blue Light	Red Light	Far-Red Light	Sunlike	FAO/WHO Model
Essential amino acids						
Val	3.48 ± 0.03 e	3.84 ± 0.03 c	4.40 ± 0.07 a	4.09 ± 0.13 b	3.68 ± 0.09 d	5.00
Met	2.25 ± 0.02 c	2.38 ± 0.03 c	2.55 ± 0.04 b	2.25 ± 0.06 c	2.77 ± 0.14 a	2.20
Ile	2.71 ± 0.02 e	3.01 ± 0.03 c	3.35 ± 0.04 a	3.13 ± 0.06 b	2.86 ± 0.14 d	4.00
Leu	4.69 ± 0.00 d	5.10 ± 0.08 c	5.91 ± 0.03 a	5.50 ± 0.06 b	4.65 ± 0.07 d	7.04
Lys	4.43 ± 0.00 d	4.85 ± 0.05 b	5.09 ± 0.10 a	4.62 ± 0.16 c	4.74 ± 0.11 bc	5.44
Phe	2.88 ± 0.02 e	3.14 ± 0.00 c	3.47 ± 0.07 a	3.25 ± 0.06 b	2.97 ± 0.05 d	2.80
Thr	3.16 ± 0.00 e	3.43 ± 0.05 c	3.73 ± 0.07 a	3.40 ± 0.13 b	3.36 ± 0.12 d	4.00
Nonessential amino acids						
Asp	5.44 ± 0.00 d	6.06 ± 0.03 c	7.22 ± 0.20 a	6.67 ± 0.13 b	5.88 ± 0.16 c	
Tyr	1.49 ± 0.05 c	1.69 ± 0.03 b	1.53 ± 0.07 c	1.38 ± 0.00 d	1.97 ± 0.07 a	
Ser	2.76 ± 0.00 c	3.12 ± 0.06 b	3.13 ± 0.00 c	2.90 ± 0.08 d	3.30 ± 0.12 a	
Glu	10.73 ± 0.11 b	11.50 ± 0.16 a	10.16 ± 0.08 c	9.52 ± 0.03 d	11.63 ± 0.3 a	
Pro	2.70 ± 0.07 e	2.99 ± 0.05 c	3.38 ± 0.10 a	3.13 ± 0.00 b	2.86 ± 0.08 d	
Gly	3.17 ± 0.02 c	3.50 ± 0.06 b	3.76 ± 0.10 a	3.48 ± 0.07 b	3.39 ± 0.09 b	
Ala	4.04 ± 0.05 d	4.51 ± 0.00 b	4.84 ± 0.10 a	4.46 ± 0.09 b	4.26 ± 0.11 c	
His	1.29 ± 0.03 c	1.49 ± 0.03 a	1.44 ± 0.10 ab	1.31 ± 0.11 bc	1.41 ± 0.05 abc	
Arg	2.85 ± 0.09 c	3.48 ± 0.05 a	4.09 ± 0.1 b	3.84 ± 0.13 c	3.35 ± 0.07 c	
UAA/TAA	40.26	39.90	38.18	38.34	39.89	
EAA/NEAA	68.47	67.16	72.06	71.52	65.78	60.00
EAA/TAA	40.64	40.18	41.88	41.70	39.68	35.38

Note: (1) UAA: umami taste amino acids; (2) TAA: total amino acids; (3) EAA: essential amino acids; (4) NEAA: nonessential amino acids.

**Table 7:** Evaluation of the composition of essential amino acids in the proteins of *P. eryngii* fruiting bodies with different light quality treatments.

Amino Acids	White Light	Blue Light	Red Light	Far-Red Light	Sunlike
Thr	69.60	76.80	88.00	81.80	73.60
Val	102.27	108.18	115.91	102.27	125.91
Met	67.75	75.25	83.75	78.25	71.50
Ile	66.62	72.44	83.95	78.13	66.05
Leu	81.43	89.15	93.57	84.93	87.13
Phe	102.86	112.14	123.93	116.07	106.07
Lys	79.00	85.75	93.25	85.00	84.00
EAAI	80.16	87.36	96.43	88.60	85.71

Note: EAAI: essential amino acid index.

**Table 8:** Measured power of LED lamps with different light qualities at 10 μmol.m^−2^. s ^−1^.

Light Quality	Measured Power/W
White light	4.4
Blue Light	4.3
Red light	2.4
Far-red light	9.9
Sunlike	4.4

## Data Availability

Not applicable.
